# Vocal Imitations of Non-Vocal Sounds

**DOI:** 10.1371/journal.pone.0168167

**Published:** 2016-12-16

**Authors:** Guillaume Lemaitre, Olivier Houix, Frédéric Voisin, Nicolas Misdariis, Patrick Susini

**Affiliations:** Equipe Perception et Design Sonores, STMS-IRCAM-CNRS-UPMC, Institut de Recherche et de Coordination Acoustique Musique, Paris, France; Max Planck Institute for Human Cognitive and Brain Sciences, GERMANY

## Abstract

Imitative behaviors are widespread in humans, in particular whenever two persons communicate and interact. Several tokens of spoken languages (onomatopoeias, ideophones, and phonesthemes) also display different degrees of iconicity between the sound of a word and what it refers to. Thus, it probably comes at no surprise that human speakers use a lot of imitative vocalizations and gestures when they communicate about sounds, as sounds are notably difficult to describe. What is more surprising is that vocal imitations of non-vocal everyday sounds (e.g. the sound of a car passing by) are in practice very effective: listeners identify sounds better with vocal imitations than with verbal descriptions, despite the fact that vocal imitations are inaccurate reproductions of a sound created by a particular mechanical system (e.g. a car driving by) through a different system (the voice apparatus). The present study investigated the semantic representations evoked by vocal imitations of sounds by experimentally quantifying how well listeners could match sounds to category labels. The experiment used three different types of sounds: recordings of easily identifiable sounds (sounds of human actions and manufactured products), human vocal imitations, and computational “auditory sketches” (created by algorithmic computations). The results show that performance with the best vocal imitations was similar to the best auditory sketches for most categories of sounds, and even to the referent sounds themselves in some cases. More detailed analyses showed that the acoustic distance between a vocal imitation and a referent sound is not sufficient to account for such performance. Analyses suggested that instead of trying to reproduce the referent sound as accurately as vocally possible, vocal imitations focus on a few important features, which depend on each particular sound category. These results offer perspectives for understanding how human listeners store and access long-term sound representations, and sets the stage for the development of human-computer interfaces based on vocalizations.

## 1 Introduction

How would you be able to describe the sound of this new exciting electric car (quiet, but not completely silent) to your grand parents? Whereas musicians, sounds engineers, or acousticians rely on a specialized vocabulary [1], most lay persons struggle to describe their auditory experience [2, 3]. Consider for example this excerpt of a conversation from a game-like experiment: a first person was describing a sound he had just heard (the “referent sound”) to a second person who, in turn, had to retrieve the referent sound from a list of distractors [4]:

- “It sounds like as if you would take a piece of corrugated cardboard. First you scrape it, then you tear it off and it sounds like Rrrrrrrr off the cardboard. You see? First, Ffffff, and then Rrrrrrrr.

- Oh I see: Ffffff then Rrrrrrr.”

The referent sound had in fact been created by unrolling a roll of kitchen paper (“Fffff”) and detaching a towel off the roll (“Rrrr”). After having struggled for a while to provide a verbal description of the sounds, the two persons finally resolved to vocalize the sounds. And it worked: the second person was able to retrieve the referent sound even though the verbal descriptions mismatched the actual source of the sounds (corrugated cardboard instead of kitchen paper). We replicated this example with several persons and confirmed that human speakers spontaneously rely on vocalizations and gestures to describe sounds whenever they run out of words. Following our previous work [4–7], the goal of the present study was to further characterize what human vocal imitations can communicate about. More precisely, it studied how well listeners can identify what vocal imitations refer to.

In fact, imitative behaviors have been reported in birds and cetaceans and are widespread in humans [8–10]. Imitations are extremely important during infants’ development and learning of skills, customs, and behaviors [11–13], and occur in all sorts of situations in adults. For instance, a person’s motor behavior (postures, mannerisms, facial expressions, phonetic features, etc.) may unintentionally match those of another person she interacts with [14–17]. Regarding *vocal* imitations, the more skilled imitators are adults: professional artists specialize in impersonating famous characters [18], and most adults can replicate a melody with a good accuracy [19, 20], thus suggesting that vocally imitating sounds is skill honed through experience [10].

These examples can be described as the reproduction of some behavior produced by a system (e.g. the vocal apparatus of one person) with another similar system (the vocal apparatus of another person). As such, imitating a behavior can be conceived as retrieving the motor parameters necessary to produce the outcome of the system [21] (but see Mercado et al., 2014 for a discussion [10]). However, the kitchen paper exemplifies a different phenomenon: reproducing some features of the outcome of a system (e.g. a kitchen paper roll) with another, different system with totally different input parameters: the voice. This immediately raises the question of whether convincingly reproducing non-vocal sounds with vocalizations is actually possible and frequent.

The short answer is yes. First, human beatboxers can replicate complex drum beats and other instrumental sounds very convincingly [22, 23]. Then, an important piece of evidence comes from linguistics. Although the most commonly agreed-upon view is that the relationships between signifier and signified (i.e. words and their meaning) is arbitrary [24], spoken languages also contain numerous instances of iconic or indexical relationships, wherein the sound of a word is perceptually evocative of its meaning. Onomatopoeias (standardized words that mimic the sound of the object they refer to), ideophones (words that evoke sounds, movements, colors, or shapes by means of a similarity between the sound of the word and the idea it refers to), and phonesthemes (sublexical units referring to higher level attributes of meaning, e.g., “gl”, as in “glitter”, “glow”, “gleam” etc. relates to “vision” and “light”) are classical examples of words evoking some aspects of non-human sounds and other sensory impressions (collectively denoted as sound symbolism) [25–32]. A classical example of sound symbolism is the “takete-maluma” phenomenon, in which subjects match nonsense words such as “takete” to images of unfamiliar angular objects and words such as “maluma” to rounded objects [33, 34]. In the auditory domain, many drumming traditions use nonsense syllables to name drum sounds, and Patel and Iversen (2003) have shown that even listeners unaware of the naming system can match the syllables and the corresponding drum sounds [35]. Iwasaki et al. (2007) also showed that English and Japanese listeners rated similarly a set of Japanese ideophones for manners of laughing and walking on a set of semantic scales [36]. Several studies have also shown that sound symbolism may help children acquire the meaning of novel action verbs, even cross-linguistically [37]. Several authors have further argued that spoken languages may have evolved from a protolanguage based in vocal and gestural imitations of environmental events [38–41].

Onomatopoieas, ideophones, and phonesthemes are *words* (or parts of words), and as such are constrained by what they refer to, but also by the linguistic system they belong to. Borrowing the words of Rhodes (1994), they are “tame” imitations, as opposed to “wild” imitations such as those reported in the kitchen paper example [42]. In comparison, wild vocal imitations have been less studied than onomatopoeias. In one such recent study, the participants’ task was to vocalize a pair of antonym adjectives (attractive/ugly, bad/good, big/small, etc.) with wild imitations [41]. Listeners could successfully match the vocalizations to the corresponding adjectives. In our previous work, we have shown that listeners categorize wild vocal imitations in the same way as they categorize the referent sounds they imitate [5], that they identify a referent sound more accurately when it is described with a vocal imitation than with a verbal description [6] and outlined different vocal strategies used to reproduce different acoustic features of the referent sounds [7].

Because of the ubiquity and effectiveness of wild and tame vocal imitations, several technical applications use them as an input method (e.g., for sound quality evaluation [43], sound retrieval [44–47]). In particular, the idea of using vocal imitations as “sketches” in the context of *sound design* has received sustained attention during the last few years [48, 49]. For example, electric cars make little noise and are sometimes augmented with artificial sounds whose goal is to warn other road users and to communicate about the car manufacturer’s identity [50]. Whereas sketching is a very important step in activities such as interaction design, graphical design, and architecture, fostering interaction and creativity, *sound* designers lack appropriate sound sketching tools. Creating tools that transform vocalizations (especially wild, unconstrained vocal imitations) into sound sketches is therefore a promising idea [48].

However, these potential applications rely on the hypothesis that these imitations can successfully convey the meaning of what they imitate. The voice is well adapted to produce and control monophonic pitch, dynamic nuances, and timing (such as in singing), as well as spectral resonances (the characteristic formants of vowel sounds) and different onset times (consonants). Many acoustic phenomena are, however, very difficult (or even impossible) for untrained imitators to produce with the voice: polyphony (yet polyphonic singing exists [51, 52]), layering of simultaneous different events, arbitrary spectral envelopes, etc. It seems therefore unlikely that a vocal imitation, even if it effectively communicates the referent sound it imitates, would do so by faithfully reproducing all the features of the referent sounds. At a more theoretical level, the implication of the effectiveness of vocal imitations to convey non-vocal sounds is that humans may possess the ability to match some features of *any* sound to their own motor vocal system.

The goal of the current work was therefore to assess to what extent wild vocal imitations can reproduce non-vocal everyday sounds. In our previous work, we had evaluated the *similarity* between the imitation and the referent sound [6]. In this study, our aim was to go beyond similarity and to assess whether listeners could identify what vocal imitations refer to *without hearing the referent sounds*. In other words, the goal of this work was to study the semantic representations evoked by simply listening to vocal imitations of sounds.

The article first describes the selection and creation of the sets of stimuli used in the experiment: the referent sounds (recordings of sounds of manufactured products and basic mechanical interactions), auditory sketches, and a set of vocal imitations performed by ten randomly drawn lay vocal imitators.

It then reports the core of the study: an experiment using a yes-no paradigm: participants were provided with verbal descriptions of a target category and simply indicated whether different sounds could correspond to the target category. We used target sounds (sounds belonging to the target categories) and distractor sounds: sounds that did not correspond to the target categories but had a similar morphological profile. This method thus measures the listeners’ ability to discriminate between target and distractor sounds (the discrimination sensitivity index d’). The analysis compared the sensitivity index for vocal imitations produced by human imitators to computational *auditory sketches* computed on the basis of sparse mathematical representations of the referent signals [53]. These sketches are scalable (i.e. the sketching procedure specifies the number of coefficients used in the representation) and used different amounts of degradation. Comparing the sensitivity indices for the different imitators to the auditory sketches allowed us to scale the effectiveness of the imitations to convey the sounds they imitate. We expected that the different imitators could be more or less proficient at producing different imitations. We therefore compared the different imitators.

Next, we assessed the acoustic features subserving the communication ability of the auditory sketches and the vocal imitations. Correlation analyses compared the relationships between sensitivity indices and different acoustic distances. In fact, auditory sketches are created by selecting the most energetic components of the sounds, thus minimizing the *overall* distance between the sketch and the referent sound (i.e. taking into account all the sound characteristics). Alternatively, human vocal imitations may *select only a few* important features, while discarding other irrelevant features: vocal imitations may focus only on those features that are important for identification of a particular sound and that can be made by the human voice. This comparison thus highlighted what drives the identification of the different stimuli.

## 2 Creating the stimuli

The study used three types of stimuli. First, we selected a number of recordings of sounds produced by manufactured products and basic mechanical interactions: the *referent sounds*. Second, we recorded *vocal imitations* of these referent sounds performed by human imitators. Third, we created *auditory sketches* using an algorithm that synthesizes a sparsfied spectro-temporal representation of the referent sounds. The selection of referent sounds and the recording of vocal imitations was part of a larger project (http://skatvg.iuav.it, last retrieved on October 13, 2016). The following paragraphs summarize the selection of referent sounds, recording of vocal imitations, and the creation of auditory sketches. Data are available at https://zenodo.org/record/57468#.V4T1a6uM67A (last retrieved on November 4, 2016).

### 2.1 The referent sounds

The goal of the selection of referent sounds was to sample across sounds occurring in an everyday environment. This selection explored two families of sound sources: *basic mechanical interactions* and *manufactured products*. These two families are not mutually exclusive. For instance the sound of hammering can be categorized as a tool or as a basic hitting action. Rather, they represent two different, overlapping points of view.

#### 2.1.1 Two points of view and two families of sounds

The first family of referent sounds consisted of sounds made by *basic mechanical interactions*. Such sounds are produced by mechanical actions acted upon an object (or substance), or by several objects in interaction: a hit on a board, the friction of a wheel on the ground, aerodynamic turbulences, water dripping, etc. Based on our previous work [3, 54, 55], the selection of basic mechanical interactions balanced an equal number of sounds produced by solid objects, liquids, and gases, and an equal number of sounds produced by discrete and continuous interactions.

In addition, many sounds that surround us are produced by human-made artefacts and machines, and consist of complex combinations of different interactions. Therefore, the second family of sounds focused on the sounds of *manufactured products*. The literature in product sound quality highlights three types of sources for such sounds: road vehicles, cars, motorcycles, buses, etc. (see for instance [56–58]), domestic appliances (refrigerators, air-conditioning, etc. see for instance [59–61]) and alarm sounds [62–64]. The selection of referent sounds therefore sampled through these categories.

#### 2.1.2 Selection of the referent sounds

The sound of manufactured products and mechanical interactions were selected from commercial or freely available databases (Hollywood Edge, Blue Box, Sound Ideas, Freesound, etc.), as well as from our previous studies [3, 54, 55, 65]. The selection of referent sounds followed three criteria.

First, the selection balanced sounds across different *morphological profiles* [66]. Morphological profiles represent the temporal evolution of a sound’s acoustic properties. Typical examples of morphological profiles are ascending or descending pitches. Our previous work showed that temporal evolution of sounds is a feature of sound imitations [7]. Thus, the selection balanced discrete and continuous sounds. Discrete sounds were further subdivided in impulsive sounds (very short sounds with a sharp burst of energy at the beginning) and sequences of sounds with a slow onset (a short sound with a more gradual buildup). Continuous sounds were further subdivided in stationary sounds (sounds whose statistical properties do not change across time) and complex sounds (sounds made of a combination of multiple events at different time scale and evolving in time). There was therefore a total of four morphological profiles.

Second, the selection balanced articulatory mechanisms that imitators may use. Previous work highlighted two main types of mechanisms in vocal imitations: myoelastic vibrations producing tonal vocalizations, and turbulences caused by constrictions of the various parts of the vocal tract and producing noisy vocalizations [67]. Unsurprisingly, imitators use myolestic vocalizations to imitate referent tonal sounds and turbulent vocalizations to imitate noisy referent sounds. Most basic mechanical interactions are noisy [3, 54]. In fact, tonal sounds are not very common in nature outside of animal vocalizations. We selected one mechanical interaction with a strong tonal component: a bell being hit and ringing. Tonal sounds are the hallmark of manufactured products that include rotating elements (engines, gears, etc.). Therefore, the selection of manufactured products balanced tonal and noisy sounds.

Third, a pilot identification experiment selected two exemplars in each category that were very well identified to their category.

The 16 categories (eight categories for the mechanical interactions and eight categories for the manufactured products) are reported in Tables 1 and 2. The selection resulted in a total of 32 referent sounds (two exemplars for each category).

**Table 1:** The selection of basic mechanical interactions, classified in morphological profiles. T = target category; D = distractor category. Descriptions between quotes were provided to the experimental participants (see Section 3). *Sounds with a strong tonal component.

Morphological profile		Category	Description
Discrete	Impulsive	Shooting (T)	Shooting, an explosion.
		Hitting* (D)	Tapping on a board. Ringing a bell.
	Slow onset/repeated	Scraping (T)	Scraping, grating, rubbing an object.
		Whipping (D)	The whoosh of a whip.
Continuous	Stationary	Gushing (T)	Water gushing, flowing
		Blowing (D)	Wind blowing; Blowing air through a pipe.
	Complex	Rolling (T)	An object rolling down a surface.
		Filling (D)	Filling a small container.

**Table 2:** The selection of sounds of manufactured products, classified in morphological profiles. T = target category; D = distractor category. Descriptions between quotes were provided to the experimental participants (see Section 3). *Sounds with a strong tonal component.

Morphological profile		Category	Description
Discrete	Impulsive	Buttons and switches (T)	A switch, a button, a computer key.
		Doors closing (D)	Closing a door.
	Slow onset/Repeated	Saws and files (T)	A person sawing or sanding an object.
		Windshield wipers* (D)	Windshield wipers wiping the windshield.
Continuous	Stationary	Refrigerator* (T)	A refrigerator’s hum.
		Blenders* (D)	Food processors switched on, processing food, and switched off.
	Complex	Printers* (T)	A printer or a fax printing pages.
		Revs up* (D)	Cars and motorcycles revving up.

### 2.2 The vocal imitations

The recording of vocal imitations was included in a larger project, where we recorded a large number of vocal and gestural imitations of a large variety of sounds.^1^ The following paragraphs describe the part of the recording sessions that are relevant to the current study. Whereas there exists a similar attempt at collecting a large amount of imitations of sounds using on-line procedures [68], our approach controlled precisely the recording procedure and the quality of the recordings in a laboratory setting.

The database of imitations has been registered with the French National Commission on Informatics and Liberty (CNIL 1890949). Overall, the procedure consisted for the imitators to listen to the referent sounds and record a vocal or a gestural imitation.

#### Setup

The imitators used a custom-made Max/MSP v.6.1 (Ircam/Cycling74) user interface. The imitators were seated in a double-walled IAC sound isolated booth. The setup consisted of a microphone (DPA d:fine omni), an audio interface (RME Fireface 800), a pair of studio monitors (Yamaha MSP5), and an Apple Mac Pro with Intel Dualcore 2.6 GHz, running MacOS 10.6.8 to record the vocal imitations. The audio was recorded at a sampling rate of 64 kHz, in 16 bits PCM WAV files.

#### Imitators

We randomly drew ten (five male, five female) imitators from of an original pool of fifty participants aged from 20 to 40 years old (median 28 years old) to keep the duration of the experiment tractable. The random sampling ensured that did not introduce any bias toward one or another imitator. All imitators reported normal hearing and were native speakers of French. None of them had received formal training in music, audio, dance, or theater, except for one person who was a professional actress. None on the imitators had any practice of vocal imitation or Foley artistry.

#### Procedure

The user interface allowed the imitators to listen to the referent sound, record and play back an imitation. There was a limit of five trials for each recording. Participants could record an imitation only if they had listened to the referent sound at least once.

The imitators were alone during the experiment to enable maximum creativity without being intimidated by the presence of the experimenter. They were instructed to provide an imitation in such a way that someone listening to them would be able to identify the sounds within the family. The imitators were instructed not to use any conventional onomatopoeia. The order of the sounds on the interface was randomized for each imitator. The experimental interface presented all referent sounds of a family and imitations on the same interface, so that the imitators could compare the different sounds. The imitators were strongly encouraged to compare and evaluate the quality of their imitations, and to compare their imitations with the referent sounds. We considered only the last imitation.

After the recording session, the experimenter reviewed all the recordings with the imitators and the imitators were invited to comment on their video and explain their strategy (“autoconfrontration interview” [69]).

### 2.3 The auditory sketches

We created auditory sketches based on the method proposed by Suied et al. (2013) [53]. It consists in three parts: 1. Computing a time-frequency representation of the signal inspired by models of peripheral auditory processing; 2. Selecting the most important elements of the representation based on a given criterion; 3. Inverting the representation. Based on the results of [53], we used the auditory spectrogram proposed by [70] and a simple maximum-peaking algorithm to select the most important elements of the representation (maximum-peaking creates fewer artifacts that the peak-picking method used by [53]). We used the NSL toolbox for signal representation and inversion (http://www.isr.umd.edu/Labs/NSL/Software.htm, last retrieved on September 15, 2015). To produce the auditory spectrogram, the acoustic signal is analyzed by a bank of constant-Q cochlear-like filters. The output of each filter is processed by a hair cell model followed by a lateral inhibitory network, and is finally rectified and integrated to produce the auditory spectrogram. The inversion of the auditory spectrogram is approximated by the convex projection algorithm proposed by [71].

**Fig 1:**
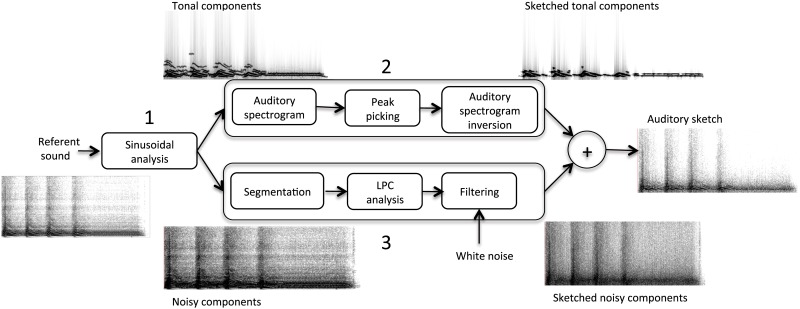
Method to create auditory sketches.

On the one hand, this method gives good results for sounds containing salient tonal contents and transients that concentrate energy in localized parts of the spectro-temporal representation, but also create audible artifacts for broadband sounds without tonal components or localized transients. On the other hand, a simple method to approximate broadband noisy signals consists of approximating the spectral envelope of the noise with the transfer function of an all-pole filter with p poles via linear predicting coding (LPC) and applying the resulting filter to a white noise [72]. Since the referent sounds that we use include harmonic sounds (e.g. electronic alarms), broadband noises (e.g. water flowing) and sounds consisting of a mix of tonal and noisy components (e.g. engines), it is important that the model can handle these types of sounds. Therefore, our method consisted in: 1. Separating tonal and noisy components; 2. Applying the method of [53] to the tonal components to create a sketch of the tonal components; 3. Applying the LPC method to the noisy components to create a sketch of the noisy components; 4. Adding the two components. This method is summarized in Fig 1.

In practice, we used Ircam’s pm2 algorithm to track the tonal components of each referent sound and separate them from the noisy components [73]. The parameters of the pm2 algorithm were adjusted for each referent sound to ensure good separation of tonal and noisy components. Then, considering tonal components only in the 0–4 kHz range, we computed the auditory spectrogram with a 8-ms frame length, a 128-ms time constant and 128 filters between 90 and 3623 Hz (referent sounds were first downsampled to 8 kHz before entering the model).

The other parameter of the tonal model was the number of coefficients per second used in the maximum-picking algorithm. We adjusted this parameter to produce sketches with different qualities. For instance, the complete auditory spectrogram uses 16000 coefficients per seconds. As a starting point, we used 4000 coefficients per second (Q3, 25% of the complete auditory spectrogram). Pilot tests showed that this produces sketches that remain well identifiable. We also created two other sketches with lower quality by dividing the number of coefficients by 5 at each step, with 800 coefficients per second (Q2, 5% of the complete auditory spectrogram) and 160 coefficients per second (Q1, 1% of the complete auditory spectrogram).

We used the same method for sketching the noisy components. However, the quality of the sketched noisy components is controlled by two parameters: the temporal resolution (hop size) and the number of LPC coefficients. As a starting point we used 36 LPC coefficients and a 9-ms temporal resolution (i.e. 4000 coefficients per second), which produced reasonable sketches for most sounds. Just as the maximum-picking method selects portions of the auditory spectrograms by sampling both the temporal and frequency dimensions, we decided to decrease the temporal resolution and the number of LPC coefficients equivalently: we multiplied the temporal resolution and divided the number of LPC coefficients by $\sqrt{5}$ between each step of quality (to distribute the fivefold division of coefficients equivalently between the noisy and tonal components). In practice, this amounted in using 16 LPC coefficients and a 20-ms temporal resolution (Q2, 800 coefficients per second), and 7 LPC coefficients and a 44-ms temporal resolution (Q1, 160 coefficients per second). The parameters are summarized in Table 3. The segmentation used an overlap of 75% whatever the temporal resolution.

**Table 3:** Parameters used to synthesize the sketches.

Parameters	Q1	Q2	Q3
Coefficients per second	160	800	4000
Temporal resolution (LPC model)	44 ms	20 ms	9 ms
LPC coefficients (LPC model)	7	16	36

It is important to note that the selection of parameters is a compromise. For instance, for stationary sounds (e.g. a refrigerator hum), using a slower time resolution improves the modeling, whereas the opposite is true for sounds with a high density of events (e.g. crumpling a piece of paper). Similarly, the modeling of tonal components focuses on the 90–4000 Hz range, because most of the sounds (but not all) have their partials in this range. In consequence, this model is more effective for certain sounds than for some others. Our selection of referent sounds balancing between different morphological profiles and textures ensured that we addressed all different cases for which the sketching method will be more or less effective.

Finally the tonal and noisy components were added while preserving the same energy ratio between the two components as in the referent signals.

## 3 Identification experiment

### 3.1 Method

All participants provided written consent to participate in this study. This study was approved by the Institutional Review Board of the French National Institute of Medical Research (CEEI-IRB Inserm 15-2160).

#### 3.1.1 Stimuli

We used the 32 referent sounds, 320 imitations (10 imitators × 32 referent sounds), and 96 auditory sketches (3 qualities × 32 referent sounds) within the eight categories of product sounds and eight categories of mechanical interactions. Half of the categories were used as targets, half as distractors. For each target, we selected the distractors with the same morphological profile, to maximize difficulty: for example, we reasoned that it would make little sense to use a sound with a stationary morphological profile (e.g. water gushing) as a distractor for a impulsive sound (e.g. shooting). Thus, only comparisons within the same morphological profile can be considered as challenging for the participants. The selected categories are represented in Tables 1 and 2.

All sounds were equalized in loudness so as to be played at 72 phones, using the algorithms provided on http://www.genesis-acoustics.com/en/loudness_online-32.html (last retrieved on August 27, 2014).

#### 3.1.2 Participants

A total of forty-nine French speaking persons volunteered as participants in the experiment. Twenty-five participants were assigned to the product sound family. One participant was excluded from analysis because his performance was at chance level for the referent sounds. This resulted in twenty-four participants in the analysis (eight male, 16 female), between 19 and 44 years of age (median 23 years old). The participants reported no hearing impairment. Twenty-four other participants (seven male, 17 female), between 20 and 50 years of age (median 24.5 years old) were assigned to the interaction family. Half of the participants identified the imitations first, the other half the sketches first.

#### 3.1.3 Apparatus

The sounds were played with an Apple Macintosh MacPro 4.1 (Mac OS X v10.6.8) workstation with a RME Fireface 800 sound card over a pair of Yamaha MSP5 studio monitors. Participants were seated in a double-walled IAC sound-isolation booth at Ircam.

#### 3.1.4 Procedure

The participants were split in four groups, two for each family (product sound or interaction). Within each family one group identified the imitations first and one group identified the sketches first (see below).

The main procedure used a yes/no paradigm. A sound was presented at each trial with a description of the target category and the participants indicated whether they felt that the sound corresponded to the description. The participants did not receive the descriptions of the distractor categories and were not aware of what were the distractor sounds. Formally, the experiment can be considered as four discrimination tasks, each consisting of discriminating the target from the distractor categories for the four morphological profiles. There were 12 trials per morphological profile ({2 target exemplars + 2 distractor exemplars} × 3 repetitions, see below). There were a total of 672 trials, resulting from 12 trials × 4 morphological profiles × {1 referent version, 10 imitations, 3 sketches}.

**Fig 2:**
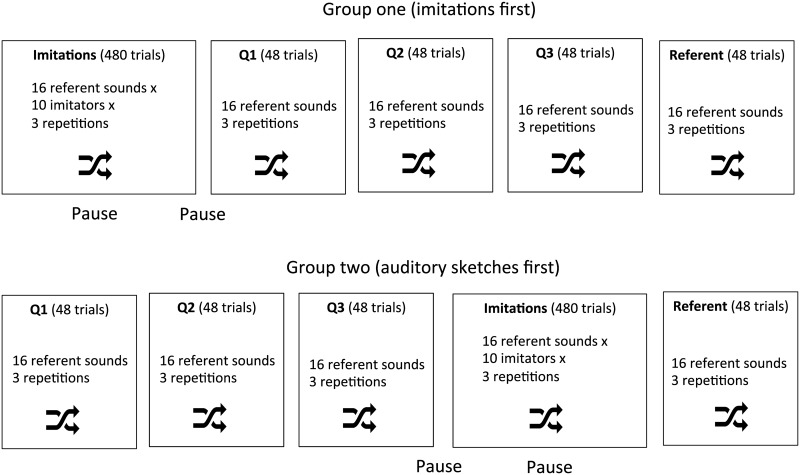
Structure of the identification experiment.

We used a blocked design with five blocks (one block for the vocal imitations, one block for each quality of auditory sketch, one block for the referent sounds). To control for the possibility that the identification of imitations could be influenced by the presentation of the auditory sketches and vice versa, we used two orders, and presented the block of the referent sounds always at the end of the session. Half of the participants started with the vocal imitations, half with the auditory sketches. The auditory sketches were always presented in order Q1, Q2, Q3. The order of the sounds in each block was also randomized. There was a pause between the blocks of imitations and the blocks of auditory sketches, and within the block of vocal imitations. Each sound was presented three times. The three repetitions were played at a different levels: baseline level (72 phones), five and ten decibels below baseline. Note that we used these the different levels only to ensure enough repetition and variability in each task. The design therefore does not allow analyzing the effect of the level. The structure of the blocks is represented in Fig 2.

### 3.2 Analysis: bias and sensitivity

We measured the sensitivity index *d*′ and the bias *ln*(*β*) with 12 trials for each morphological profile [74] (the sensitivity index measures the ability to discriminate the stimuli, and the bias measures the tendency to answer one or the other response more systematically). We used the procedure proposed by [75] to account for perfect discrimination (several participants perfectly discriminated several referent sounds). With this method, *d*′ = 2.93 corresponds to perfect discrimination.

#### 3.2.1 Bias

One initial hypothesis was that participants could be biased toward or against vocal imitations, because vocal imitations can clearly be perceived as originating from a human vocalization, whereas referent sounds and sketches are clearly identifiable as resulting from a mechanical phenomenon and some electronic processing. In a yes/no paradigm, *ln*(*β*) is a measure of the tendency for a participant to be biased toward responding more often “yes” than “no” (and vice versa), regardless of what the correct answer is. The quantity *ln*(*β*) equals zero when there is no bias, is positive when a participant is biased toward the “yes” answer (liberal bias) and negative when the participant is biased toward the “no” answer (conservative bias). It ranges from minus infinity (the participant systematically responds “no”) to plus infinity (the participant systematically responds “yes”).

Biases were overall small: they ranged from -1.07 to 1.07 across participants, morphological profiles, and types of sounds, with a median of 0. The average bias was 0.02 for the three sketches, -0.17 for the referent sounds, and 0.17 for the ten imitators. Paired t-tests showed that these differences were statistically significant (referent sounds vs. sketches t(46) = 4.37, p<.01, referent sound vs. imitations, t(46) = -9.07, p<.01). These results indicate that participants were in fact more liberal for the vocal imitations than for the referent sounds. One interpretation (based on the participants’ comments) is that they were more tolerant for the imitations precisely because they expected them to be less precise than the referent sounds or the sketches.

#### 3.2.2 Sensitivity: global analysis

The index of discrimination sensitivity *d*′ measures the ability to discriminate the stimuli (i.e. categorize them as matching the category label or not). The index is null when the participants respond randomly (hit rate equals false alarm rate), positive when they provide more correct than incorrect answers, and negative when they provide more incorrect than correct answers (i.e. invert their responses). The *d*′ were submitted to a four-way analysis of variance (ANOVA) with the Family (product sounds, interactions) and the Order of the blocks (imitations first or auditory sketches first) as between-participant factors, and the Type of sound (the 10 imitations, Q1, Q2, Q3, and referent sound itself) and the Morphological profile (impulse, repeated, stationary, complex) as within-participant factors. The sphericity assumption was tested using Mauchly tests. When necessary, the degrees of freedom were adjusted with Geisser-Greenhouse correction to account for violation of the sphericity assumption. All p-values are reported after this correction. For the sake of clarity, we also report the *unbiased* percentages of correct identification (*upc*) in addition to the *d*′ values, computed by transforming the *d*′ values assuming no bias (i.e. false alarms = 1 − hit rate).

The results of the ANOVA show that there was no significant main effect of the Order of the blocks (F(1,44) = 0.33, p = .566), and that it did not interact with the Family (F(1,44) = 0.83, p = .367) nor with the Type of sound (F(13,572) = 0.42, p = .925) or the Morphological profile (F(3,132) = 0.70, p = .522). The three-way interactions (between the Order, the Families, and Morphological profile; between the Order, the Families, and Types of sounds; between the Order, the Morphological profile and Type of sounds) were not significant (respectively F(3,132) = 1.83, p = .157; F(13,572) = 0.70, p = .711; F(39,1716) = 1.01, p = .450), nor was the four-way interaction between the four factors (F(39,1716) = 1.33, p = .161). The order of the blocks will therefore not be considered in the following. There was no main effect of the Family (F(1,44) = 2.25, p = .141), which means that there was no overall difference of performance between the two families of sounds. There was a significant interaction between the Family and the Type of sounds (F(13,572) = 14.75, p<.01), between the Family and the Morphological profile (F(3,132) = 25.80, p<.01). The three-way interaction between the Family, the Type of sound and the Morphological profile was also significant (F(39,1716) = 8.38, p<.01).

The main effect of the Type of sound was significant (F(13,572) = 65.99, p<.01). Tukey HSD tests showed that the performance (averaged across all the other factors) were overall not significantly different between imitators, except for the difference between the imitator who elicited the worst performance (“worst imitator” I32, *d*′ = 0.77, p<.01) and the two imitators who elicited the best performances (“best imitator” I23, *d*′ = 1.25; I20, d’ = 1.20), and between the best and the second worst imitator (I12, *d*′ = 0.87, p<.05). The differences between imitations and sketches are detailed below. The main effect of the Morphological profile was significant as well (F(3,132) = 32.45, p<.01). Discrimination sensitivity was best for the impulsive morphological profiles (*d*′ = 1.72, upc = 85.6%), followed by the repeated (*d*′ = 1.35, upc = 79.2%), the stationary (*d*′ = 1.01, upc = 72.5%), and the complex morphological profiles (*d*′ = 0.79, upc = 67.9%). The two-way interaction between the Type of sound and the Morphological profile was also significant (F(39,1716) = 12.20, p<.01).

To interpret these different significant interactions, we analyzed the *d*′ values separately for each family and morphological profile, resulting in eight separate analyses.

#### 3.2.3 Sensitivity: breakdown of results

**Fig 3:**
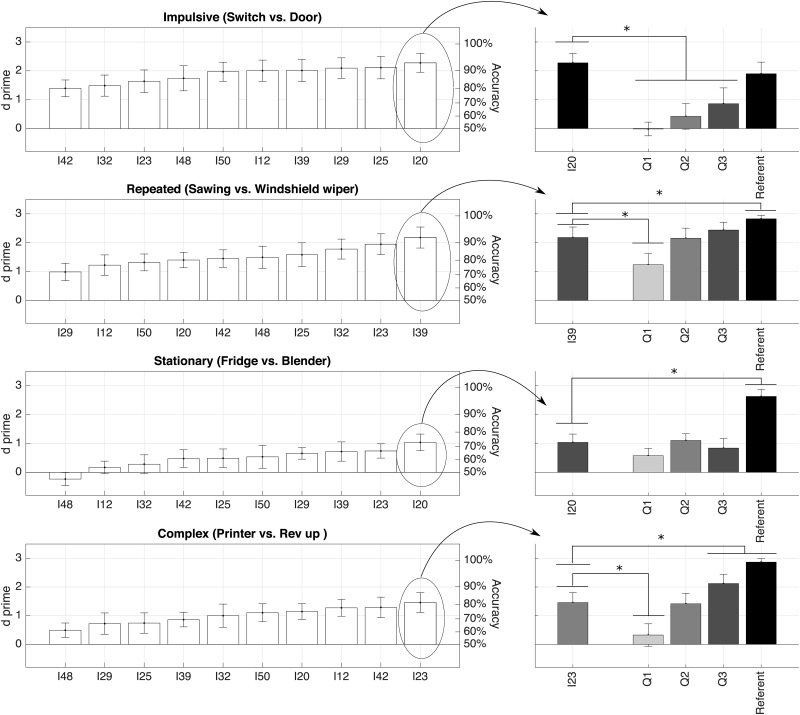
Discrimination sensitivity indices (*d*′) and accuracy (assuming no bias) for the four morphological profiles in the family of product sounds. The left panels represent the data for the ten imitators (I32, I23, etc. are the code of each imitator). The right panels zoom on the best imitation for each morphological profile and compare it to the three auditory sketches and the referent sounds. Gray shadings represent the quality of the sketches (from light gray—Q1—to dark gray—Q3—and to black—referent sound). The right panels also represent the results of four t-tests comparing the best imitator to each of the three auditory sketches and the referent sounds. When the best imitation is not significantly different from an auditory sketch (with an *alpha*-value of.05/4), it receives the same shading. Vertical bars represent the 95% confidence interval of the mean. *significantly different from chance level after Bonferroni correction (p<.05/4).

**Fig 4:**
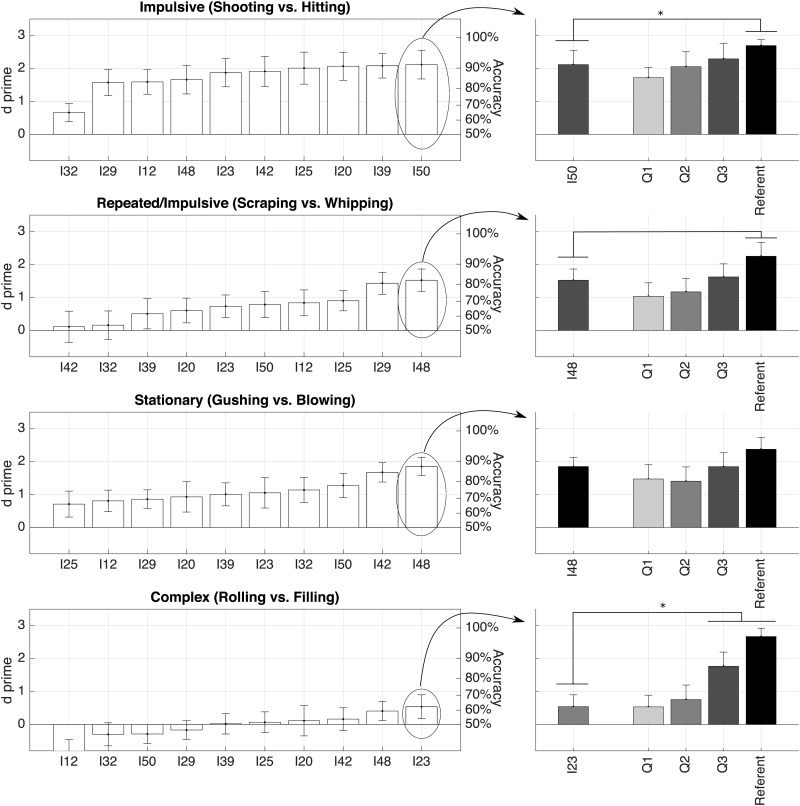
Discrimination sensitivity indices (*d*′) and accuracy (assuming no bias) for the four morphological profiles in the family of basic mechanical interactions. See Fig 3 for detail.

Analyses were conducted separately for the two families (product sounds and interactions) and the four morphological profiles in each family (impulsive, repeated/slow onset, stationary, complex). For each morphological profile, the left panels of Figs 3 and 4 first represent the sensitivity indices of the ten imitators, sorted from worst to best. Then the right panels of these figures focus on the best imitators for each morphological profiles, and represent the results of four t-tests comparing the sensitivity index for the best imitators to the three auditory sketches and the referent sounds.

Fig 3 shows that the patterns of results are different for the four morphological profiles of product sounds. The referent sounds were accurately identified for the four morphological profiles. Performance with the imitations was overall good for the *impulsive* morphological profiles (i.e. switches vs. doors), with sensitivity ranging from *d*′ = 1.4 (upc = 80%) to *d*′ = 2.3 (upc = 93%). In this case, the best imitation (I20) lead to discrimination sensitivity that was not significantly different from the referent sound (*d*′ = 1.9, upc = 88%). Performance was also good for the *repeated* profiles (sawing vs. windshield wipers), with sensitivity ranging from *d*′ = 0.99 (upc = 72%) to *d*′ = 2.2 (upc = 92%). The best imitation (I39) was not statistically different from Q3 (*d*′ = 2.4, upc = 95%). Performance was worse for the *stationary* morphological profiles (refrigerators vs. blenders), with sensitivity ranging from *d*′ = -0.23 (upc = 45%) to *d*′ = 1 (upc = 73%). In fact, performance was also not very good the four sketches, and the best imitation (I20) was not statistically different from Q3 (*d*′ = 0.84, upc = 69%). Performance was better for the *complex* morphological profiles (printers vs. revs up), with sensitivity ranging from *d*′ = 0.49 (upc = 61%) to *d*′ = 1.5 (upc = 81%). Performance for the best imitator (I23) was not significantly different from Q2 (*d*′ = 1.4, upc = 80%).

Regarding the sounds of interactions, Fig 4 shows the referent sounds were accurately discriminated. Sensitivity was high for the imitators of the *impulsive* profiles, ranging from *d*′ = 0.66 (upc = 65%) to *d*′ = 2.1 (upc = 91%). The best imitator (I50) was not significantly different from Q3 (*d*′ = 2.3 upc = 94%). Performance was worse for the *repeated/slow-onset* profiles, with sensitivity ranging from *d*′ = 0.11 (upc = 52%) to *d*′ = 1.5 (upc = 82%). The best imitator (I48) was not significantly different from Q3 (*d*′ = 1.6 upc = 84%). Performance was better for the *stationary* profiles, with sensitivity ranging from *d*′ = 0.71 (upc = 66%) to *d*′ = 1.8 (upc = 88%). The best imitator (I48) was not significantly different from the referent sound (*d*′ = 2.4 upc = 95%). Discrimination was not accurate for the *complex* profiles, with sensitivity ranging from *d*′ = -0.96 (upc = 28%) to *d*′ = 0.54 (upc = 62%). The best imitator (I23) was not significantly different from Q2 (*d*′ = 0.76 upc = 67%).

## 4 What makes a good imitation?

The previous analyses show that some imitations could be identified as successfully as the best auditory sketches (and in some cases the referent sounds themselves) in all cases but two (sounds of refrigerators and of a rolling object). Whereas this shows that it is *possible* to vocally imitate the referent sounds effectively, it also indicates a gradient of effectiveness. The analyses described below compared the acoustics of the imitations, auditory sketches, and referent sounds to determine what makes some imitations better identified than some others.

### 4.1 Acoustic distance between target and distractor sounds

Although the participants never directly compared target and distractor sounds, the experimental paradigm is formally a discrimination task. For each morphological profile, the participants sorted the stimuli into two categories (target vs. distractor). As such, the ability to discriminate the two stimuli may be related to acoustic distance between the stimuli in target and distractor categories: the larger the acoustic distance between the two categories, the easier the discrimination.

Defining a generic (all-purpose) acoustic distance between two arbitrary waveforms is an eluding question in audio signal processing. Here we tested two methods: one distance based on the alignment cost of auditory spectrograms (“auditory distance”), and one distance based on a set of generic acoustical features averaged across the duration of the signals (“feature distance”).

The first method adapted the model of auditory distance created by Agus et al. (2012) [76] and used by Isnard et al. (2016) [77]. Originally, this model uses the time-frequency distribution of energy for each sound, estimated using spectro-temporal excitation patterns (STEPs) [78] that simulate peripheral auditory filtering. Auditory distances are then computed by aligning pairs of STEP times series using a dynamic time-warping algorithm. The cost of alignment is used as the distance between two sounds. Here, we used auditory spectrograms instead of STEPs, consistent with the method used to create the auditory sketches. Such a distance is however sensitive to the duration of the sounds, and distances can only be compared for sounds with the same duration. Therefore, signals were first time-stretched to the same duration before computing the distances, using a phase vocoder algorithm to preserve spectral information [79]. The result of this procedure is to scale the auditory distances to the same range for the different sounds. Otherwise, very short sounds (e.g. impulsive morphological profiles) result in smaller distances overall (their are fewer time steps to align) than longer sounds (e.g. stationary or complex morphological profiles). All sounds were time-stretched to 4.6 s (the average duration of all stimuli). Distances were normalized so that a unit auditory distance corresponds to the distance between a 4.6-s white noise and a 4.6-s 1-kHz pure tone. As expected, the auditory distances represent very well the transformations operated by the auditory sketches: the correlation between the logarithm of the auditory distances and the logarithm of the number of coefficients per second is r = -0.99 for the product sounds and r = -1.00 for the sounds of mechanical interactions.

The second method used a set of features developed for the classification of vocal imitations [80]. These features are based on the temporal evolution of 13 standard features. Three features, represent the duration and sparseness of the signal: the number of active regions, the absolute duration, and the relative duration. Seven features correspond to standard audio features: the median (across time) of the noisiness (ratio of noise over harmonic energy), zero-crossing rate, pitch strength (the saliency of the pitch sensation), pitch, loudness, and the standard deviation (across time) of the pitch and the spectral centroid. Pitch and pitch strength were computed with the *swipep* algorithm [81]. The other features were computed with the *IrcamDescriptor Toolbox* [82]. The three last features were specifically developed for the project. The envelope of the amplitude and the main spectral peak of the signals were first modeled by a two-piece linear regression. The first two features are then calculated as the slope of the second piece of the model for the amplitude and the spectral peak. The third feature corresponds to the goodness of fit between the model and the amplitude [80]. The values of these features were first standardized, so that the distributions of features all have the same unit standard deviation and zero mean. The feature distance between two sounds was then computed by taking the Euclidean norm of the difference of the two vectors (e.g. Euclidean distance in the feature space).

As expected, the two distances are related, though not identical. The correlation between the two distances, calculated over the 14 types of sounds and four yes/no morphological profiles (four possible distances) was r = 0.63 (N = 224, p<.01) for the product sounds and r = 0.32 (N = 224, p<.01) for the interaction sounds.

Analyses (using auditory distances) showed however that the correlation between sensitivity indices and the target-distractor distance was weak and inconsistent for both methods. With the auditory distances, the coefficient of correlation was statistically significant and positive only for gushing/blowing (r = 0.69, N = 14, p<.01), and shooting/hitting (r = 0.64, N = 14, p<.05). For the other morphological profiles, coefficients of correlation were not significantly different from zero and varied from r = -0.40 to r = +0.36. With the feature distances, coefficients of correlation were significant and positive for gushing-blowing (r = 0.59, N = 14, p<.05), significant and negative for the printer/wipers (i.e. contrary to our expectation r = -0.56, N = 14, p<.05), and ranged from -0.19 to +0.38 for the other morphological profiles.

### 4.2 Acoustic distance to the referent sound

Another interpretation of the results is that performance may be related to the acoustic distance between each sound (sketch or imitation) and the referent sound it refers to. Even if participants never heard the referent sounds until the end of the experiment, they may have compared each sound to some mental representation of the category, corresponding closely to the referent sounds selected for each category (the referent sounds were very well identified to each category).

**Fig 5:**
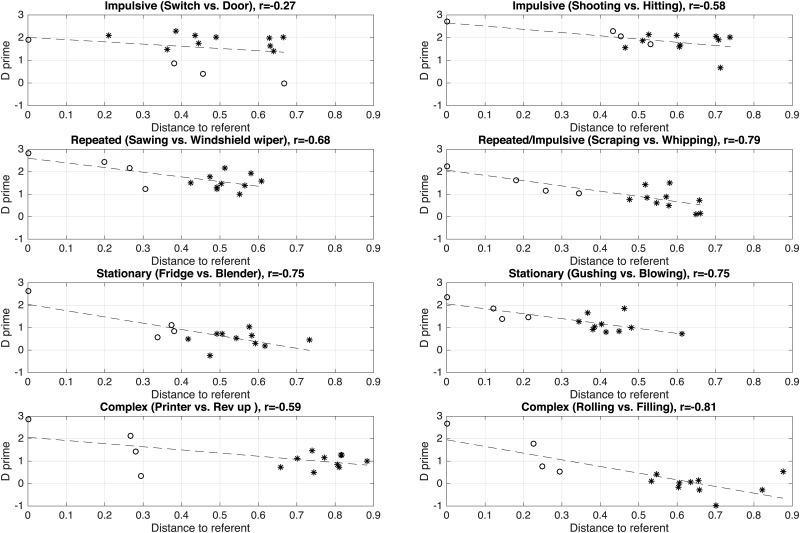
Indices of discrimination sensitivity (*d*′) as a function of the auditory distance between each sound and its corresponding referent sound. Auditory differences are calculated by computing the cost of aligning the auditory spectrograms of the two sounds [76]. Circles represent the referent sounds (in this case, the distance is therefore null) and the three sketches. Black stars represent the ten imitators. The dashed line represents the regression line between the auditory distances and the *d*′ values.

Fig 5 represents the sensitivity indices *d*′ as a function of the auditory distances, for the two families of sounds and the four morphological profiles. For most morphological profiles, there is a clear trend for the sensitivity index to decrease with the auditory distances. We measured the coefficient of correlation to estimate the strength of this association (with 14 data points and an *α*-value of.05, the threshold of significativity is r = 0.53, and with an *α*-value of.01, the threshold of significativity is r = 0.66). For instance, there is a clear correlation between *d*′ and the auditory distances for the *repeated* morphological profiles for both families (r = -0.68, p<.01 for the product sounds, r = -0.79, p<.01 for the interactions), as well as for the *stationary* morphological profiles (r = -0.75, p<.01 for both families). The correlation is also good for the *complex* morphological profiles in the interaction family (r = -0.81, p<.01). However the auditory distances do not predict the sensitivity in some cases. For instance, for the *impulsive* product sounds (“shooting”), the auditory distance between the referent sounds and the imitations is large (which should result in poor performance) whereas the sensitivity is also large.

**Fig 6:**
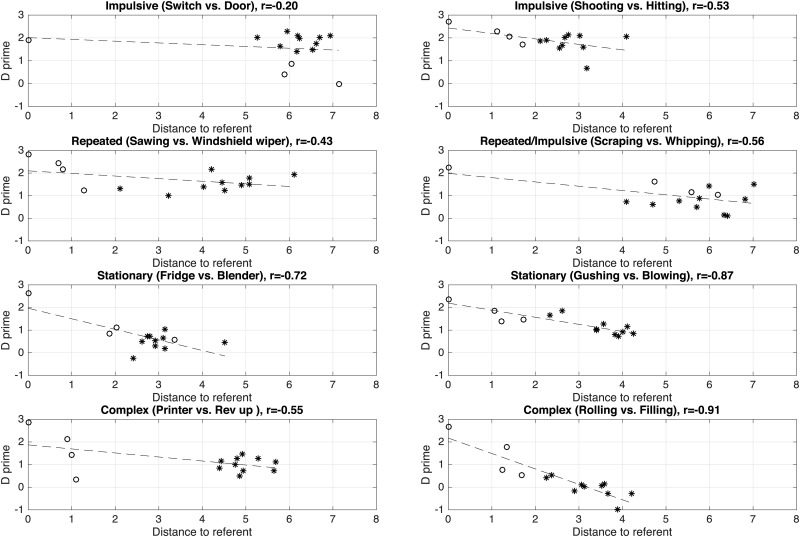
Indices of discrimination sensitivity (*d*′) as a function of the feature distance between each sound and its corresponding referent sound. Feature differences are calculated by computing the Euclidean norm of the features defined by [80].

Fig 6 represents the sensitivity indices *d*′ as a function of the feature distances, for the two families of sounds and the four morphological profiles. Overall, correlations are worse than with the auditory distances, even though the patterns look overall similar.

### 4.3 Phonetic overview

To further analyze why certain vocal imitations are well identified and some others are not, we listened to the two best- and two worst-identified imitations for each morphological profile and reported a description of their characteristic elements in Table 4. These descriptions are the results of a consensus between three of the authors, using annotations of the vocal imitations made by trained phoneticians, using a system specifically developed for the database of imitations [67].

**Table 4:** Phonological description of the best and worst vocal imitations. The last column suggests the cues that are important for successful identification. M = Morphological profile; I = impulse; R = repeated; S = stationary; C = complex; SO = slow onset.

M	Best imitations	Worst imitations	Cue to identify
Product sounds			
I	Two rapid clicks	One single click or two slow clicks	Two rapid clicks
R	Rhythm of egressive & ingressive streams and fricatives	Irregular sequence of trills and fricatives	Regular repetition egressive & ingressive streams + fricatives
S	Continuous voiced part with (modulated) fricatives; initial occlusion	Continuous egressive stream with initial occlusion + fricatives	Voiced part, low *f*_0_ + fricatives
C	Sequence of voiced and fricative parts + egressive & ingressive streams	Unstructured sequence of egressive streams + voiced and fricative parts	Structured voiced + fricative parts
Interaction			
I	Short occlusion + decreasing egressive stream and fricatives	Occlusion + egressive stream with trill or fricative parts	Short occlusion + decreasing turbulent stream + fricatives
SO	Alternate or modulated fricative parts (+ trills)	Egressive stream with some fricatives, rhythm is irregular	fricatives + regular rhythm
S	Egressive stream with fricatives with fine regular texture	Fine texture with timbre variations	Fricatives + fine regular texture
C	Continuous breathy voiced part with trills or fricatives, or sequence of trills & fricatives, with increasing pitch or spectral centroid	Sustained voiced note; sequence of clicks	Fricatives + trills + spectral increase

Some cases are easy to interpret. For the *discrete* morphological profiles (impulsive and repeated) in both families, the *duration and timing* of the elements composing the sounds appears to be critical, regardless of the acoustic quality of each element. For example, the best-identified imitations of switches were made of a rapid succession of two clicks, whereas the worst-identified imitations were made of only one click, or a slower succession of two clicks. The best-identified imitations of shooting sounds created a sharp contrast between an initial loud explosion (created by an initial occlusion of the lips and a sudden release of an airstream) and a quieter sustained turbulent noise imitating the reverberation. Similarly, for the repeated morphological profiles (for both families), the rhythmic alternation of ingressive (e.g. breathing in) and egressive (e.g. breathing out) turbulent airstreams created a convincing imitation of the sawing action, whereas a looser or less audible rhythm made the imitations more difficult to identify.

In contrast, *voicing and fundamental frequency* (*f*_0_) appear to play an important role for *stationary* morphological profiles such as the sounds of the refrigerator. What make imitations of the *complex morphological* profiles successful or not is however more difficult to interpret.

These observations suggest that what makes an imitation identified or not is related to an iconic similarity between the imitation and the referent sounds. By iconic similarity, we mean that the imitation reproduces certain characteristic of the sounds (e.g timing, fundamental frequency), whereas ignoring some other characteristics (e.g. timbre). What characteristic is important seem to depend on each particular sound. Therefore, any acoustic distance measure that takes into accounts every sound features without weighing them cannot systematically predict how well a given imitation will be perceived.

### 4.4 Discussion

Despite the differences between the two distances, the same patterns emerge from the results: whereas there is a clear trend for the auditory sketches (performance decreases as the acoustic distance between the sketch and the target increases), this trend does not generalize very well to the vocal imitations. Overall, the acoustic distances between the vocal imitations and the referent sounds are much larger than the distances between the sketches and the referent sounds, yet the performance can be equivalent or better for the vocal imitations compared to the auditory sketches. In short, acoustic distances to the referent sounds do not predict performance very well for the vocal imitations.

This may result from two possible phenomena: one possibility is that the acoustic distances do not fully capture the “perceived” difference between the sounds. The auditory distance is an effective measure for the referent sounds and the sketches, because all these sounds share the same structure: the sketches are simplified versions of the referent sounds. But the vocal imitations are structurally different. They are vocal sounds whereas the referent sounds are non-vocal. Another possibility is that identification of vocal imitations is based on more complex mechanisms than simply evaluating the acoustic distance between a given vocal imitation and a referent sounds. For instance, iconicity may come into play, as suggested by the phonetic overview of the best and worst imitations. In this view, the process of creating a vocal imitation of a referent sound does not only amount in vocalizing a sound as similar as possible as the referent sound. Whereas the vocal imitations (and especially the best identified ones) do sound similar to the referent sounds, this similarity seems to be based on a few selected, category-specific features, that are picked up by the imitators.

## 5 General Discussion

The experiment measured to what extent the sensory representations elicited by a sound (sketch or imitation) matched the memory representations elicited by a descriptive label of the sound categories (target and distractor). Overall, the results show that vocal imitations discriminated accurately targets and distractors for most categories of sounds, and thus matched fairly well to the representations of these categories.

In fact, participants were even *biased* toward the vocal imitations: they were more inclined to associate a vocal imitation with a target label (independently of whether it truly corresponded to the target) than the auditory sketches. One reason could be that they expected them to be less precise than the referent sounds or the sketches, and thus were more tolerant, precisely because they produced by humans. A similar effect has been reported for pitch judgments: listeners are more inclined to call a note in-tune when it is sung than when it is played with a non-vocal timbre [83].

Even when considering *unbiased* sensitivity indices, our results show that most imitators produced imitations that listeners could match with the different sound categories well above chance level. The comparison with the auditory sketches allowed us to further quantify this performance. We had created three levels of quality: Q3 (4000 coefficients per second), Q2 (800 coefficients per second), and Q1(160 coefficients per second). The highest quality level (Q3) corresponds to a relatively weak degradation. For instance, using a similar algorithm, Suied and Isnard found that listeners could discriminate basic emotions and broad categories of sounds (singing voices, birds, musical instruments) above chance level for sketches made of as few as 10 coefficients per second, and reached the same performance as the referent sounds for 1000 coefficients per second [53, 77]. In our data, discrimination sensitivity followed the number of coefficients per second in most cases (although sketches resulted in poor performance for the refrigerator sounds, independently of the number of coefficients). The sketches are therefore a useful scale to compare the imitators with. Although the task used in the current experiment was much more difficult and thus required more coefficients per second (the distractor sounds were very similar to the target sounds, and the target and distractor categories corresponded to a subordinate level of specificity compared to Suied and Isnard), there were systematically several imitations that elicited performance similar to the highest-quality sketches, or the referent sounds themselves in some cases. This shows that, for most categories of sounds, it is *possible* to produce vocal imitations that evoke very accurately the category they imitate and are not confounded with nearby distractor categories.

The best imitations were not systematically produced by the same imitators: the “best imitator overall” (the person who produced the imitations that elicited the best performance across the different morphological profiles and families of sounds) was only significantly different from the two “worst imitators overall”, but not significantly different from all the other imitators. The differences of performance observed across the different sounds were therefore not due to some imitators being more skilled *in general*, but rather to the fact that each imitator would be effective only for certain sounds. This suggests that successfully imitating the different sounds requires reproducing different features for each sound, and that each imitator may be proficient with vocalizing only certain features. In fact, listening to the imitations shows that each imitator may have preferentially used a limited number of vocal techniques for imitating the sounds: for instance, we noticed that one person would often whistle, another person would often use noisy expirations, etc. The autoconfrontation interviews also suggested that, after a few trials, the imitators had created a small set of personal vocal techniques that they felt comfortable with, which they would reuse and combine during subsequent imitations. In this interpretation, an imitator would create a successful imitation whenever a referent sound is consistent with the particular techniques favored by this imitator. Because the imitators had absolutely no vocal training, their repertoire was rather limited, and specialized toward certain types of sounds. It is however remarkable that even untrained, randomly-drawn imitators can produce such accurate imitations. Previous work has shown that a good proportion of untrained singers can imitate simple melodies accurately (but not precisely) [20], and that untrained imitators can reproduce the rhythm and spectral centroid of artificial sounds as accurately as professional singers [7], but little else is known about the ability to vocally imitate other features of non-vocal, non-musical sounds. This result is nevertheless particularly encouraging, because it shows that even completely untrained imitators can produce imitations that are easily identifiable in most cases. On-going work is currently studying how to train imitators and improve the effectiveness of imitations [84].

Looking in more detail at the results shows that performance strongly depended on the morphological profiles. Performance was very good for some morphological profiles (e.g. impulsive profiles). For the sounds of shooting, for instance, performance was high both for the vocal imitations and auditory sketches. This probably results from discrimination being overall easier. Impulsive sounds were different from all the other sounds because of their very short duration. Furthermore, the target sounds (shooting) were different from the distractors (hitting) because of their long reverberation tail, which was clearly conveyed by the vocal imitations. Results are more interesting when there is a difference of performance between the sketches and the vocal imitations. For instance, for the sounds of switches (the distractors consisting of door sounds), performance was high for the vocal imitations, but weak for the sketches. On the one hand, the imitators constantly imitated these sounds by clicking their tongue or lips, which produced very short high-pitched sounds clearly distinguishable from the imitations of the door sounds used as distractors (imitations contained much lower frequencies). On the other hand, the auditory sketches could not accurately reproduce the abruptness of the sounds, because of the temporal resolution of the processing. An other example is that of the sound of a small object rolling down a ramp (the distractor sound being the sound of filling a vessel). Whereas the auditory sketches resulted in fair performance, most vocal imitations could not be discriminated above chance level, and some imitations were even systematically matched with the wrong sounds (i.e. negative *d*′). Both target (rolling) and distractor sounds (filling) consisted of long noisy sequences whose identity is conveyed by the complex and rapid pattern of micro events overlapping in time and frequency (e.g. the repetition of random impacts for the rolling sound and the evolution of the spectrum for the filling sound). Whereas these patterns were preserved by the sketching procedure, their complexity proved to be too difficult to reproduce with the voice.

All these results point toward the idea that vocal imitations are effective when the referent sounds are characterized by the evolution in time of a few features that can be reproduced with the voice: duration, repetition and rhythm, pitch, noisiness, etc. When the sounds are characterized by complex patterns of many overlapping elements, vocal imitations cannot be effective (but auditory sketches can).

The analyses of the correlations between discrimination performance and distances between sounds support this interpretation. A usual finding in studies using a similar paradigm is that discrimination performance is correlated with the acoustic distance between target and distractor categories (see for instance [77]). This was however not the case here. Instead, discrimination performance was correlated with the distance *between test sounds (imitations or sketches) and target referents sounds*. This strongly confirms our interpretation that the participants compared the imitations to some imagined sound inferred from the category labels. In fact, the task for the participants consisted in telling whether a sound (vocal imitation or auditory sketch) matched a category described by short label without ever hearing the referent sounds. Conceptually, participants thus had to match the sensory representation of the stimulus with the mental representations associated with a certain category indexed by lexical label [85].

The correlation analyses tested two types of acoustic distances, measuring different aspects of the sounds: *auditory distance*, sensitive to the temporal structure of the sound spectra, and *feature distance*, sensitive to statistics summarized over the sound duration. The analyses showed that identification performance could be predicted by the auditory distance better than by the feature distance. This confirms the importance of the temporal structure of the sounds for sound identification.

In more detail however, correlations were high for the auditory sketches, but coarser for the vocal imitations. The high correlation for the auditory sketches is not surprising, since both the sketching algorithm and the method used to compute the distances were based on the same auditory model. The weaker correlation for the vocal imitations calls for a more nuanced interpretation. For instance, it could be the case that the referent sounds used to generate the sketches and the imitations did not completely match the participants’ representations of the categories. In fact, in the two main theoretical views of categorization (the prototypical and the exemplar views [86]), the distance to some prototype or to the exemplars is critical to category membership: In the prototypical view, category membership is defined by the distance to some prototypes. In the exemplar view, categories are made of a collection of exemplars and defined by the similarity between the exemplars. It is therefore possible that the referent sounds that we used were not good exemplars of the categories they referred to (because the participants had never heard such a similar sound, or because they associated these sounds to some other categories). However, performance was systematically very high for the referent sounds, which rules out this hypothesis.

A more plausible interpretation is that the distance models were simply not perfectly capturing the perceived distance between the imitation and the category it refers to. In fact, the distance model considered the same properties equivalently for all sounds. But, as the previous discussion pointed out, it is possible that the features that are relevant for identifying the sounds are different for each sound category, and depend on the context in which sounds are heard. If that hypothesis is correct, it is not possible for any general purpose metric to capture the quality of an imitation, precisely because it would weigh equivalently the features that are characteristic of the sound category and the features that human listeners simply dismiss as irrelevant. For instance, for the sounds of sawing, listeners would identify imitations as long as they correctly reproduce the rhythm of the referent sound, and would not be influenced by the particular timbre of the imitation, whereas an accurate reproduction of timbre would be critical for other category. Distance models, however, would incorrectly consider imitations with a different timbre as being very distant from the referent sound, because they weigh the timbre and the rhythm equivalently. Vocal imitations have thus the potential to highlight the degree to which each feature contributes to sound identification.

Overall, these results set the stage for new exciting research. First, they offer perspectives for the development of intuitive human-computer interfaces. They show that vocal imitations do more than just providing a more or a less accurate version of the sounds they refer to: instead, they *sketch* a person’s idea of a given sound, exactly as free-hand drawing would sketch a graphical idea. As such, the results confirm that vocalizations could be used for fast and interactive sound prototyping, especially in the context of sound design where initial phases of creation are recognized as being crucial, but where fast and intuitive tools are still lacking [87]. The results also show that vocal imitations pick up the most relevant aspects of a sound category, and that these relevant features depend on each sound category and the context wherein they occur. The challenge for any human-computer interface that uses vocalizations as an input will therefore be to match such adaptive versatility.

At a more theoretical level, these results imply that humans are endowed with a mechanism that allow them to map the features of *any* sound to the motor commands necessary to reproduce these features with the voice, and not only vocal sounds. Several other species (birds, cetaceans) display imitative vocalizations of conspecifics or other species, but this ability is in fact rare [10]. It has been a matter of debate as to whether imitations result from general learning or specialized mechanisms [88]. Here we show that humans can successfully imitate a variety of non-vocal sounds, even without training and only a few trials. These results are therefore not consistent with approaches that consider that imitations are subserved by a mechanism wherein representations of actions and consequences of actions are isomorphic (the consequences of an action are encoded together with parameters of that action) [21], precisely because they do not consider the fact that human vocalizations can also successfully imitate non-vocal sounds. Instead, our results are consistent with the idea that human imitators may possess inverse auditory-vocal models capable of generating the motor commands necessary to produce a given acoustic feature [89], and that this ability is not limited to the voice [90] but may in fact apply to any sound.

Seen from another perspective, this result that imitations pick up the pieces of information most relevant for identification also holds promise for using vocal imitations as a tool to explore auditory cognition. What the successful imitations of a sound category tell the researcher is precisely what are the important features for identifying that sound category. Thus, vocal imitations could be used as an investigation method to explore the content of auditory memory. The imitations used in this study were produced while or just after the imitators were listening to the referent sounds. As such, they reflect how sounds are encoded in a short-term memory storage. Future work will explore how imitations could also reflect how sounds are coded in long-term memory. In fact, there is a current debate in auditory cognition about the nature of auditory sensory and memory representations of sound sources. On the one hand side, studies of the timbre of musical instruments have systematically reported that musical instruments are characterized by a few dimensions, systematically found across a wide range of sounds [91–93]. On the other hand, machine learning techniques that best recognize musical instruments, styles, etc. are not based on a few recurrent dimensions: they use and create whichever pieces statistical information fit a given classification task, relying on high-dimensional representations [94–97]. Another important result is that prior exposure to complex, abstract noise textures with little structure improves the performance at a subsequent repetition detection task [98]. Other results show that identifying sounds may in fact rely on diagnostic features, idiosyncratic to each sound class, learnt by listeners through their individual experience [99]. This suggests that auditory representations for noise encodes some complex statistical properties of sounds, and that these properties may in fact be noise- and listener-dependent. Studying vocal imitations of these sounds may highlight what these properties are.

## 6 Conclusion

This study investigated how listeners identify three different types of sounds: recordings of real unambiguous sounds (sounds of human actions and manufactured products), human vocal imitations, and computational “auditory sketches”. The results show that identification performance with the best vocal imitations were similar to the best auditory sketches (and even to the referent sounds themselves in some cases) for most categories of sounds, even though imitators had absolutely no vocal training. This shows that vocal imitations match the mental representations of the sounds they refer to, even when listeners have no awareness of the referent sounds imitated. Detailed analyses have further shown that the overall distance between an imitation and a referent sound does not completely account for identification performance. Our results suggest that instead of reproducing all the referent sounds’ characteristics as accurately as vocally possible, vocal imitations focus on a few important features, which depend on each particular sound category and in the context in which the sounds are presented. The result that human vocalizations are able to accurately imitate non-vocal sounds also suggests that humans are endowed with a mechanism that generates motor commands necessary to reproduce acoustic features of any type of sound (and not only vocal sounds). The ability of vocal imitations to emphasize important features for sound identification offers prospectives both for applications to sound design, but also to investigate auditory cognition.
